# Levels of blood lead and urinary cadmium in industrial complex residents in Ulsan

**DOI:** 10.1186/s40557-017-0179-7

**Published:** 2017-06-26

**Authors:** Sang Hoon Kim, Yang Ho Kim, Hyun Chan An, Joo Hyun Sung, Chang Sun Sim

**Affiliations:** Department of Occupational and Environmental Medicine, Ulsan University Hospital, University of Ulsan College of Medicine, 877, Bangeojinsunhwando-ro, Dong-gu, Ulsan, 44033 Republic of Korea

**Keywords:** Industrial complex, Environmental exposure, Lead, Cadmium

## Abstract

**Background:**

Populations neighboring industrial complexes are at an increased health risk, due to constant exposure to various potentially hazardous compounds released during industrial production activity. Although there are many previous studies that focus on occupational exposure to heavy metals, studies that focused on environmental exposure to lead and cadmium are relatively rare. The purpose of this study is to evaluate the extent of the environmental exposure of heavy metals in residents of industrial area.

**Methods:**

Four areas in close proximity to the Ulsan petrochemical industrial complex and the Onsan national industrial complex were selected to be included in the exposure group, and an area remotely located from these industrial complexes was selected as the non-exposure group. Among the residents of our study areas, a total of 1573 subjects aged 20 years and older were selected and all study subjects completed a written questionnaire. Blood and urine samples were obtained from about one third of the subjects (465 subjects) who provided informed consent for biological sample collection. Total 429 subjects (320 subjects from exposure area, 109 subjects from non-exposure area) were included in final analysis.

**Results:**

The geometric mean blood lead level among the subjects in the exposed group was 2.449 μg/dL, which was significantly higher than the non-exposure group’s level of 2.172 μg/dL. Similarly, the geometric mean urine cadmium levels between the two groups differed significantly, at 1.077 μg/g Cr. for the exposed group, and 0.709 μg/g Cr. for the non-exposure group.

In a multiple linear regression analysis to determine the relationship between blood lead level and related factors, the results showed that blood lead level had a significant positive correlation with age, the male, exposure area, and non-drinkers. In the same way, urine cadmium level was positively correlated with age, the female, exposure area, and smokers.

**Conclusions:**

This study found that blood lead levels and urine cadmium levels were significantly higher among the residents of industrial areas than among the non-exposure area residents, which is thought to be due to the difference in environmental exposure of lead and cadmium. Furthermore, it was clear that at a low level of exposure, differences in blood lead or urine cadmium levels based on age, gender, and smoking status were greater than the differences based on area of residence. Therefore, when evaluating heavy metal levels in the body at a low level of exposure, age, gender, and smoking status must be adjusted, as they are significant confounding factors.

## Background

Populations neighboring industrial complexes are at an increased health risk, due to constant exposure to various potentially hazardous compounds released during industrial production activity [1]. Ulsan, which is located multiple industrial complexes including the petrochemical industrial complex and the Onsan industrial complex, is one of the top industrial cities in Korea. For this reason, since 2012, the National Institute of Environmental Research and university of Ulsan have been carrying a study named “Monitoring of Exposure to Environmental Pollutants and Health Effects of Inhabitants in Industrial Complexes” to assess the human health impact of potentially hazardous compounds emitted from large-scale industrial complexes.

Although heavy metals exist naturally in the environment, their concentration in the soil, water, and air can increase as a result of growing industrial emission. Long-term ingestion of such contaminated food, water, and air increase a health risk, as heavy metals accumulate in the human body [2]. Although there are many previous studies that focus on occupational exposure to heavy metals [3–5], studies that focused on environmental exposure to these metals are relatively rare [6, 7].

In this study, we focused on lead and cadmium, as they are the most suited for assessing human exposure to environmental heavy metals. The main sources of general lead exposure are gasoline, industrial emissions, and food [8]. Cadmium is also absorbed by inhaling air which is polluted by environmental tobacco smoke, and industrial emissions [9]. Previous studies found that lead or cadmium accumulated over time in the body can have harmful health effects, even at low concentration. Even low level of lead, lead in body linked to lower intelligence and academic achievement in children and is associated with various adverse health effects including lowering glomerular filtration rate and increasing blood pressure in adults [10–13]. Low level of cadmium also causes tubular proteinuria and decreases bone mineral density [14–17]. Because environmental exposure to lead and cadmium can cause serious health problems as well as occupational exposure, these two heavy metals were selected as the focus of our study.

Our study utilized third year data (2014) from the “Monitoring of Exposure to Environmental Pollutants and Health Effects of Inhabitants in Industrial Complexes”. To compare the levels of environmental exposure to heavy metals between the residents of industrial area and residents in the non-exposure area who are geographically and geomorphologically separated from industrial complexes, we analyzed their blood lead levels and urine cadmium levels. Subsequently, we investigated the factors influencing the level of heavy metal accumulation in the body.

## Methods

### Study subjects

**Fig. 1:**
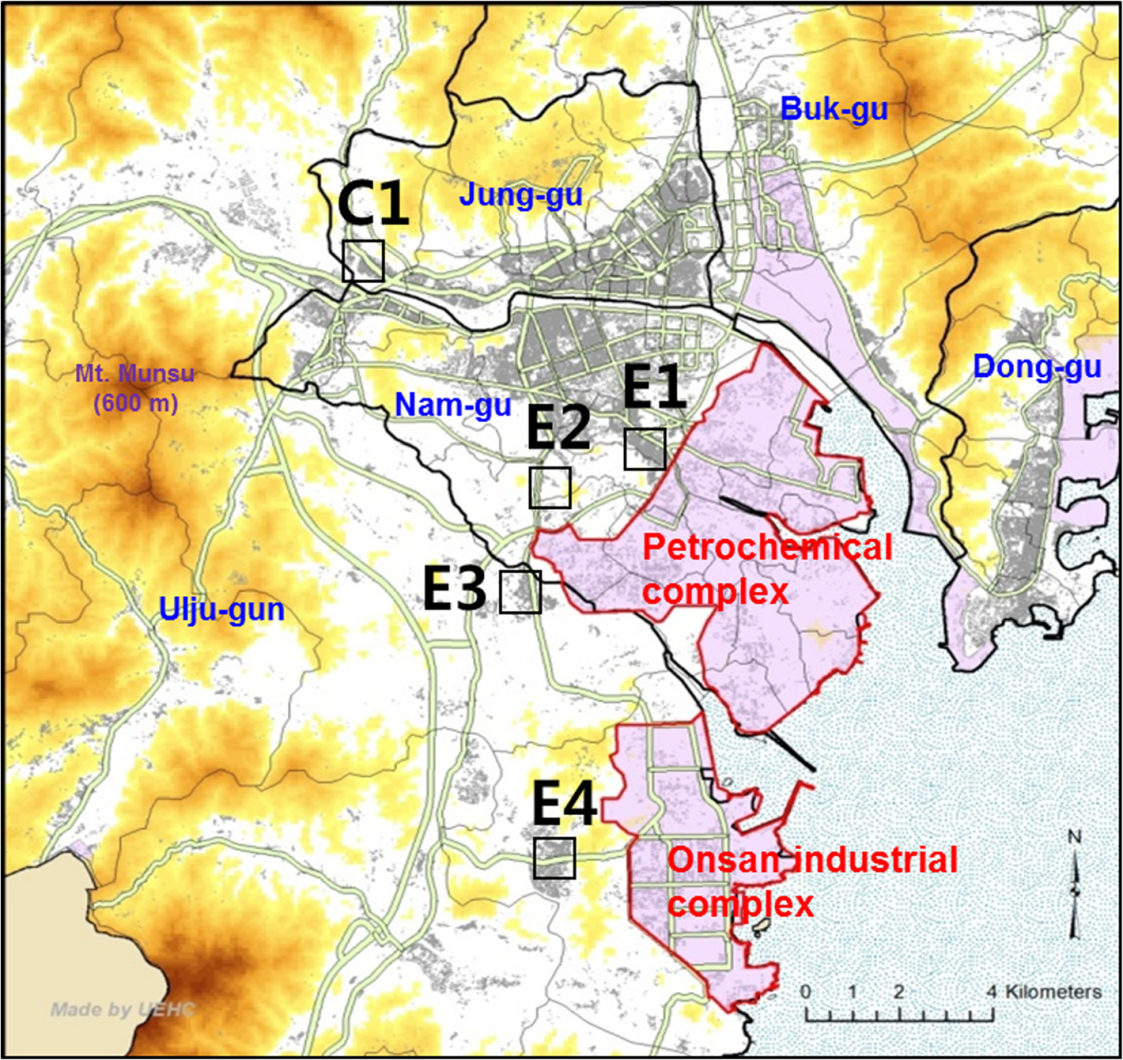
Map showing (a) location of the study areas with high (E1–4) and low (C1) exposure to air pollution. *Red shaded regions* and *gray lines* indicate industrial areas and main roads, respectively

Four areas in close proximity to the Ulsan petrochemical industrial complex and the Onsan national industrial complex (Fig. 1) were selected to be included in the exposure group (areas surrounding Yaeum-Jangsaengpo Community Service Center: E1, Gaewoon Elementary School: E2, Cheongryang Town Hall: E3, Onsan Community Service Center: E4), and an area remotely located from these industrial complexes was selected as the non-exposure group (area neighboring the Daun Elementary School: C1). Among the residents of our study areas, a total of 1573 subjects aged 20 years and older were selected, taking into account the areas’ population, gender, and age distribution. All study subjects completed a written questionnaire designed to survey demographic characteristics, environmental hazard exposure factors, lifestyle, current dietary habits, and time use patterns. Using these biological samples, 20 types of chemicals including 3 types of heavy metals, urinary volatile organic compound metabolites, polycyclic aromatic hydrocarbon metabolites, and phthalate metabolites were analyzed. And we used blood lead and urinary cadmium data in this study. Blood cadmium indicates recent exposure, but do not correlate with body burden or clinical outcome. However, urine cadmium indicates reflects integrated exposure over time and total body burden [18]. The present study considered chronic cumulative exposure to cadmium in industrial complex area.

Blood and urine samples were obtained from about one third of the subjects (465 subjects) who provided informed consent for biological sample collection. Of the collected biological samples, 21 samples were identified to be inadequate, leaving a total of 444 samples to be included (335 subjects from exposure area, 109 subjects from non-exposure area). The final statistical analysis included a total of 429 subjects, following the exclusion of 15 subjects whose raw data had many missing values.

### Laboratory methods

For the evaluation of blood lead levels, collected samples were first diluted and then analyzed with a graphite furnace-atomic absorption spectrophotometer (GF-AAS). 100 μl of whole blood, 100 μl of 0.2% HNO_3_, and 800 μl of diluted solution (0.2% (NH_4_)_2_HPO_4_ & 0.2% Triton X-100) were combined in a 5 ml tube. This tube was put in a vortex mixer, after which analysis was conducted using a GF-AAS (AA-600, PerkinElmer) [19].

A similar procedure was followed for evaluation of urine cadmium levels. Collected samples were first diluted, and then analyzed using a GF-AAS. 100 μl of urine, 100 μl of 0.2% HNO_3_, 800 μl of diluted solution (0.2% (NH_4_)_2_HPO_4_ & 0.2% Triton X-100) were combined in a 5 ml tube. The tube was put in a vortex mixer, after which analysis was conducted by a GF-AAS (AA-800, PerkinElmer). Measurements of cadmium in urine are adjusted to creatinine [20]. External quality control was provided by the German External Quality Assessment Scheme (G-EQUAS).

### Statistical analysis

Data collected from the 429 subjects (320 subjects from exposure area, 109 subjects from non-exposure area) were analyzed using SPSS 21.0 (IBM SPSS Inc., Chicago, IL). Since heavy metal levels shows a log-normal distribution, the geometric mean and confidence intervals were calculated. For comparison of the mean values, log transformed data were analyzed with an independent t-test. Distribution according to a categorical variable was analyzed using a chi-square test. In order to identify the factors influencing blood lead levels and urine cadmium levels while keeping relevant covariates constant, a multiple linear regression analysis was performed. Significance level was set at *p* < 0.05; a *p* value less than 0.05 indicated statistical significance.

## Results

Subjects included in the final analysis were between 20 and 84 years of age. The mean age of the exposed group was 51.41 ± 16.32 years, and 48.08 ± 17.17 years for the non-exposure group. The difference between the mean ages was not significant. Furthermore, the differences in smoking status, drinking status, distance between residence and a major road, and body mass index (BMI) between the two groups were not significant. However, there was a statistically significant difference in education level between the two groups (Table 1).

**Table 1:** General characteristics of exposed group and non-exposed group

		Non-exposure	Exposure	*p* value
Gender	Men	43	130	0.829
	Women	66	190	
Mean age (AM)		48.08 ± 17.17	51.41 ± 16.32	0.070
Age group	20–29	19	42	0.483
	30–39	17	35	
	40–49	23	62	
	50–59	17	66	
	60–69	20	68	
	Over 70	13	47	
Body Mass Index	Non-obese	79	227	0.759
	Obese	30	93	
Smoking status	Non-smoker	73	227	0.436
	Smoker	36	93	
Drinking status	Non-drinker	74	202	0.370
	Drinker	35	118	
Educational status	High school and less	49	214	<0.001^*^
	College and more	60	106	
Distance to road	Above 100 m	59	143	0.098
	Under 100 m	50	175	

unit: arithmetic mean ± standard deviation

*AM* arithmetric mean

^*^ *p* < 0.05

The geometric mean blood lead level among the subjects in the exposed group was 2.449 μg/dL, which was significantly higher than the non-exposure group’s level of 2.172 μg/dL. Similarly, the geometric mean urine cadmium levels between the two groups differed significantly, at 1.077 μg/g Cr. for the exposed group, and 0.709 μg/g Cr. for the non-exposure group. Significant gender based differences were found in terms of blood lead levels and urine cadmium levels. Mean blood lead level for male subjects was 2.835 μg/dL, significantly higher than that of female subjects, which measured at 2.108 μg/dL. However, mean urine cadmium level in female subjects was 1.185 μg/g Cr., was significantly higher than that of male subjects, which measured at 0.718 μg/g Cr. Both the blood lead level and urine cadmium level increased with age. This relationship was more pronounced in urine cadmium level compared to blood lead level. Blood lead level was higher significantly in the group of low level of education, drinkers, and smokers. Urine cadmium level was higher significantly in the group of low level of education, drinkers, and non-smokers. Blood lead levels and urine cadmium levels among the group of subjects whose residence was within 100 m of a major road were higher than those of who resided farther away from a major road. However, the difference was not significant. The concentration of blood lead in exposed and non-exposed area were analyzed by dividing the variables such as gender, age group, BMI, smoking status, educational level, distance to road, the blood lead concentration in the exposed area was higher than that in the non-exposure area, except for the age group of 30s and the BMI of 25 or more. In the same way, the concentration of urine cadmium in the exposed area was higher than that of the non-exposure area in all variables (Table 2).

**Table 2.** Geometric mean and range of blood lead (μg/L), cadmium (μg/g Creatinine) of study subjects

Classification variables		Number	Lead			Cadmium		
			Total	Non-exposure	Exposure	Total	Non-exposure	Exposure
				2.172 ± 1.590	2.449 ± 1.510^*^		0.709 ± 2.344	1.077 ± 2.169^*^
Gender	Men	173	2.835 ± 1.516 (2.663–3.018)	2.599 ± 1.486	2.917 ± 1.522	0.718 ± 2.221 (0..603–0.810)	0.484 ± 2.169	0.818 ± 2.149^*^
	Women	256	2.108 ± 1.484 (2.008–2.213)^*^	1.933 ± 1.600	2.173 ± 1.436^*^	1.185 ± 2.144 (1.079–1.302)^*^	0.908 ± 2.254	1.300 ± 2.063^*^
Age group	20–29	61	1.698 ± 1.450 (1.544–1.868)	1.544 ± 1.516	1.773 ± 1.413	0.373 ± 1.966 (0.313–0.443)	0.275 ± 2.169	0.428 ± 1.797^*^
	30–39	42	2.139 ± 1.404 (1.946–2.351) ^†^	2.247 ± 1.489	2.088 ± 1.364	0.638 ± 1.869 (0.536–0.759) ^†^	0.535 ± 2.112	0.695 ± 1.730
	40–49	85	2.386 ± 1.444 (2.204–2.583) ^†^	2.283 ± 1.516	2.426 ± 1.419	0.806 ± 2.043 (0.691–0.941) ^†^	0.690 ± 2.127	0.855 ± 2.007
	50–59	83	2.683 ± 1.573 (2.430–2.962) ^†§^	2.434 ± 2.751	2.751 ± 1.610	1.367 ± 1.830 (1.198–1.560) ^†§^	0.983 ± 1.826	1.488 ± 1.785^*^
	60–69	88	2.594 ± 1.467 (2.391–2.814) ^†§^	2.420 ± 1.424	2.647 ± 1.480	1.453 ± 1.872 (1.272–1.660) ^†§^	1.165 ± 1.840	1.550 ± 1.862
	Over 70	60	2.701 ± 1.604 (2.390–3.052) ^†§^	2.290 ± 2.117	2.827 ± 1.434	1.628 ± 1.826 (1.394–1.903) ^†§^	1.198 ± 1.704	1.734 ± 1.841
Body Mass Index	Non-obese (<25 kg/m^2^)	306	2.319 ± 1.543 (2.208–2.435)	1.977 ± 1.599	2.451 ± 1.502^*^	0.970 ± 2.262 (0.885–1.064)	0.687 ± 2.351	1.094 ± 2.157^*^
	Obese (≥25 kg/m^2^)	123	2.522 ± 1.509 (2.344–2.715)	2.784 ± 1.417	2.443 ± 1.532	0.964 ± 2.254 (0.834–1.115)	0.768 ± 2.347	1.038 ± 2.204
Smoking status	Non-smoker	300	2.179 ± 1.517 (2.078–2.284)	1.906 ± 1.571	2.274 ± 1.486^*^	1.027 ± 2.337 (0.933–1.131)	0.752 ± 2.496	1.136 ± 2.233^*^
	Smoker	129	2.905 ± 1.474 (2.715–3.108)^*^	2.831 ± 1.446	2.934 ± 1.487	0.844 ± 2.038 (0.746–0.956)^*^	0.628 ± 2.023	0.946 ± 1.983^*^
Drinking status	Non-drinker	276	2.476 ± 1.545 (2.351–2.607)	2.332 ± 1.538	2.531 ± 1.546	0.812 ± 2.225 (0.739–0.893)	0.595 ± 2.259	0.911 ± 2.149^*^
	Drinker	153	2.205 ± 1.503 (2.066–2.353)^*^	1.870 ± 1.652	2.315 ± 1.439^*^	1.330 ± 2.103 (1.181–1.498)^*^	1.027 ± 2.269	1.436 ± 2.023^*^
Educational status	High school and less	263	2.514 ± 1.500 (2.393–2.641)	2.314 ± 1.654	2.562 ± 1.461	1.299 ± 2.033 (1.191–1.415)	1.068 ± 1.904	1.358 ± 2.049^*^
	College and more	166	2.172 ± 1.567 (2.027–2.327)^*^	2.063 ± 1.532	2.236 ± 1.586	0.608 ± 2.122 (0.542–0.683)^*^	0.507 ± 2.360	0.675 ± 1.950^*^
Distance to road	Above 100 m	202	2.320 ± 1.538 (2.185–2.463)	2.167 ± 1.502	2.386 ± 1.550	0.944 ± 2.231 (0.845–1.055)	0.686 ± 2.452	1.077 ± 2.062^*^
	Under 100 m	225	2.422 ± 1.533 (2.290–2.562)	2.179 ± 1.695	2.497 ± 1.477^*^	0.992 ± 2.290 (0.889–1.106)	0.736 ± 2.232	1.080 ± 2.267^*^

unit: geometric mean ± standard deviation (95% confidence interval)

^*^ *p* < 0.05

^†^ *p* < 0.05 vs. age group (20–29)

^§^ *p* < 0.05 vs. age group (30–39)

The results showed that blood lead level had a significant positive correlation with age, the male, exposure area, and non-drinkers (Table 3).

**Table 3:** Multiple linear regression model of association of ln blood lead with exposure after adjusting for covariates (*n* = 429)

Independent variables	β coefficient (Standardized β coefficient)	*p* value	Model	
			R^2^	*p* value
Age (Years)	0.009 (0.363)	<0.001	0.260	<0.001
Education level (College and more vs. High school and less)	−0.023 (−0.026)	0.619		
Drinking status (Yes vs. No)	−0.144 (−0.161)	<0.001		
Smoking status (Yes vs. No)	0.081 (0.086)	0.159		
Gender (Men vs. Women)	0.198 (0.227)	<0.001		
Exposure (Yes vs. No)	0.088 (0.090)	0.039		
Distance to road (Under 100 m vs. Above 100 m)	0.014 (0.016)	0.711		

*ln* natural log

The analysis found that urine cadmium level was positively correlated with age, the female, exposure area, and smokers. The results showed that among smokers: age, the female, and exposure area had a positive correlation with urine cadmium level. A similar trend was found among non-smokers (Table 4).

**Table 4:** Multiple linear regression model of association of ln urine cadmium with exposure after adjusting for covariates

	Independent variables	β coefficient (Standardized β coefficient)	*p* value	Model	
				R^2^	*p* value
Total	Age (Years)	0.026 (0.531)	<0.001	0.480	<0.001
(*n* = 429)	Education level (College and more vs. High school and less)	−0.106 (−0.064)	0.153		
	Drinking status (Yes vs. No)	−0.029 (−0.017)	0.679		
	Smoking status (Yes vs. No)	0.261 (0.147)	0.004		
	Gender (Men vs. Women)	−0.656 (−0.396)	<0.001		
	Exposure (Yes vs. No)	0.328 (0.175)	<0.001		
Smoker	Age (Years)	0.025 (0.608)	<0.001	0.521	<0.001
(*n* = 129)	Education level (College and more vs. High school and less)	−0.162 (−0.113)	0.124		
	Drinking status (Yes vs. No)	−0.238 (−0.113)	0.085		
	Gender (Men vs. Women)	−0.444 (−0.159)	0.014		
	Exposure (Yes vs. No)	0.427 (0.270)	<0.001		
Non-smoker	Age (Years)	0.027 (0.520)	<0.001	0.468	<0.001
(*n* = 300)	Education level (College and more vs. High school and less)	−0.054 (−0.031)	0.584		
	Drinking status (Yes vs. No)	0.009 (0.005)	0.914		
	Gender (Men vs. Women)	−0.706 (−0.318)	<0.001		
	Exposure (Yes vs. No)	0.284 (0.144)	0.001		

*ln* natural log

## Discussion

Environmental exposure to lead has been decreasing, as consumption of leaded gasoline has declined over the years [21]. However, lead is still present in household heating fuel and in anti-knock agents for automobiles. Cadmium is found in tobacco smoke, food, lubricating oil, and tires, and it is released into the atmosphere during the processes of combustion and wear and tear in tires. Most of the studies on these two metals focused on occupational exposure, studies focused on environmental exposure were relatively rare.

We surveyed a total of 429 adults (aged 20 years and older) residing in the areas neighboring industrial complexes or the non-exposure area in Ulsan. The blood lead levels and urine cadmium levels of subjects residing in the exposure area (areas neighboring industrial complexes) were higher than those in the non-exposure group. This result suggests that the concentration of heavy metals of soil and atmosphere in industrial area is higher than that in non-exposure area [22]. Furthermore, the geometric mean blood lead level (2.449 μg/dL) of the exposure group, as well as that (2.172 μg/dL) of the non-exposure group was higher than the mean of 1.77 μg/dL reported by the 2012 Korean National Environmental Health Survey (KNEHS). Similarly for mean urine cadmium level, both the exposure group and the non-exposure group showed a higher measurement than the mean of 0.664 μg/g Cr. reported by the KNEHS. In addition, It was higher than the geometric mean of blood lead level (1.91 μg/dL) and urine cadmium level (0.61 μg/g Cr.) reported by the 2008 Korea National Survey for Environmental Pollutants in the Human Body. These findings indicate that environmental pollution in Ulsan is more severe than in other regions, due to the heavy metals released by its multiple industrial complexes.

The overall geometric mean of blood lead was 2.835 μg/dL in men and 2.108 μg/dL in women. This is higher than reported in the recent US National Health and Nutrition Examination Survey (NHANES) (1.09 μg/dL in 2011–2012) for subjects aged 20 years and older [23]. Differences may be due to control of industrial emission, earlier phasing out of leaded gasoline, and tighter public health measures in USA. The urine cadmium levels in our subjects were higher than reported in the recent US NHANES (0.220 μg/g Cr in 2011–2012) for subjects aged 20 years and older [23]. Differences may be due to control of industrial emission, and tighter public health measures in USA. In addition, rice consumption which was the main source of cadmium exposure was higher in Korea [24]. Moon et al. [24] studied the general population of South Korea and found that diet was the main source of cadmium exposure, but cigarette smoking is also a well-known source of cadmium exposure [25].

In addition to age, gender, smoking status, and drinking status, which have already been identified by previous studies to be associated with blood lead level, we incorporated exposure status (industrial area vs. non-exposure area) and distance between residence and a major road in the multiple linear regression analyses. These analyses were performed while keeping relevant covariates constant. The results showed that blood lead level increased with age, and was higher in males and smokers; consistent with findings reported by previous studies [26, 27]. It is thought that the positive correlation between age and blood lead level may be attributed to a cumulative effect, owing to the long half-life of heavy metals in human body [28, 29]. Meanwhile, the higher blood lead level among the male subjects may be explained by the fact that in the female body, lead is deposited in the marrows due to the action of female hormones [30]. Notably, at a low level of exposure, age and gender differences in blood lead levels were greater than the differences resulting from subjects’ degree of exposure (residence) to lead. However, analysis performed to identify the correlation between blood lead level and distance between residence and a major road (<100 m vs. ≥100 m) did not reveal any significant correlation. Urine cadmium level also increased with age, and was higher in females and smokers, consistent with the findings of previous studies [31]. It is interesting that there was a contrast in findings between urine cadmium levels and blood lead levels based on gender. The positive correlation between urine cadmium level and the female gender is thought to be due to the fact that cadmium retention is generally higher in women than in men [32], and that iron deficiency more commonly observed in women accelerates cadmium absorption in the intestines [33, 34]. It is notable that the age-dependent increase in urine cadmium levels is more drastic than the age-dependent increase in blood lead levels. Since smoking has a significant influence on urine cadmium levels [35], an analysis was performed which separate smoking and non-smoking subjects. In both groups, a higher level of urine cadmium was measured among older subjects, female subjects, and those who reside in exposure area. As both lead and cadmium are present in tobacco smoke, a positive correlation is inferred between smoking and both blood lead and urine cadmium levels [36]. However, living near the big roads did not affect blood lead and urine cadmium levels. Ulsan industrial complex is a very old industrial complex, and it will have a long and lasting influence on environmental pollution. Indeed, the results of the measurement of hazardous air pollutants showed that environmental lead and cadmium levels in exposed areas are higher than those in unexposed areas in our study. The effect of environmental pollution around the industrial complex was apparently identified by comparing blood lead and urine cadmium levels of people in the exposed area with those in the non-exposed area. In addition, the present study showed that individual factors such as age, gender, and smoking were found to affect blood and urine metal levels more than environmental exposure does. Thus, at a low level of exposure, differences in blood lead or urine cadmium levels based on age, gender, and smoking status were likely to be greater than the differences based on area of residence.

The strength of our study lies in minimization of confounding factors through stratified sampling according to age for selection of our subjects to both the exposure and non-exposure groups. Beginning with this study, it is thought that various follow-up studies will be possible by using cumulative data of “Monitoring of Exposure to Environmental Pollutants and Health Effects of Inhabitants in Industrial Complexes”.

The present study has some limitations. First, it was not able to evaluate dietary exposure to lead and cadmium, which is one of the primary sources of heavy metal exposure. Nevertheless, because food consumption is not closely linked to residence, it is thought that exposure due to dietary habit will not lessen our study’s validity. Second, we defined the exposure group and non-exposure group according to subjects’ places of residence. Unfortunately, this overlooked the subjects’ employment sites, where a great deal of time is spent on a daily basis. Nonetheless, because we ruled out respondents suspected of occupational exposure based on questionnaire, it is thought that its confounding effect is limited.

## Conclusions

We analyzed and compared the blood lead levels and urine cadmium levels of subjects residing in the industrial complex areas of Ulsan and those residing in geographically and geomorphologically separate non-exposure areas of Ulsan. In addition, we examined which factors affect blood lead levels and urine cadmium levels. This study found that blood lead levels and urine cadmium levels were significantly higher among the residents of industrial areas than among the non-exposure residents, which is thought to be due to the difference in environmental exposure of lead and cadmium. Furthermore, the present study showed that at a low level of exposure, differences in blood lead or urine cadmium levels based on age, gender, and smoking status were greater than the differences based on area of residence. Therefore, when evaluating heavy metal levels in the body at a low level of exposure, age, gender, and smoking status must be adjusted, as they are significant confounding factors. We hope that the results of this study will provide the groundwork required for future research studies and policy decisions relevant to environment health in the Ulsan area.

## Abbreviations

AM: Arithmetic mean
BMI: Body mass index
GF-AAS: Graphite furnace-atomic absorption spectrophotometer
GM: Geometric mean
KNEHS: Korean National Environmental Health Survey
ln: natural log
NHANES: National Health and Nutrition Examination Survey
